# Sensing Deformation in Vacuum Driven Foam-Based Actuator via Inductive Method

**DOI:** 10.3389/frobt.2021.742885

**Published:** 2021-12-14

**Authors:** Seonggun Joe, Hongbo Wang, Massimo Totaro, Lucia Beccai

**Affiliations:** ^1^ Soft Biorobotics Perception Lab, Istituto Italiano di Tecnologia (IIT), Genova, GE, Italy; ^2^ The BioRobotics Institute, Scuola Superiore Sant’Anna, Pontedera, Italy

**Keywords:** vacuum-powered soft actuator, proprioceptive artificial muscle, soft sensing, porous material, robust adaptive control

## Abstract

Perception in soft robotics is crucial to allow a safe interaction to effectively explore the environment. Despite the inherent capabilities of soft materials, embedding reliable sensing in soft actuators or robots could introduce constraints in the overall design (e.g., loss of deformability, undesired trajectories, etc.) or reduce their compliant characteristics. Consequently, an adequate stiffness for both sensor and actuator becomes a crucial design parameter. In particular, for sensing the deformation related to actuation motion, sensing and actuating strategies must work in full mechanical synergy. In this view, an inductive sensing solution is presented, exploiting open-cell foam and a copper (Cu) wire in an Inductive Foam Sensor (IFS). Due to entangled air cells high deformability is enabled upon vacuum pressure, and proprioceptive information is provided. The IFS is then successfully integrated into the earlier developed Ultralight Hybrid Pneumatic Artificial Muscle (UH-PAM), which encases an elastomeric bellow skin and plastic rings. Such sensorized UH-PAM (SUH-PAM) is capable of a high contraction ratio (54% upon −80 kPa), while the inductive sensing shows a high sensitivity of 0.01031/1% and a hysteresis of 5.35%, with an average error of 1.85%, respectively. In order to implement a robust feedback control system, an adaptable proportional sliding mode control is presented. As a result, the SUH-PAM motion can be controlled to the mm-scale, with an RMSE of 0.925 mm, and high robustness against disturbances is demonstrated.

## 1 Introduction

In recent years, research in soft robotics addressed completely novel solutions for achieving tasks that could not be addressed by robots built from rigid bodies. Remarkable results were demonstrated including locomotion, manipulation, etc., for a range of applications like e.g., medical devices, and wearable systems, due to safe and versatile functionalities (Walker et al., 2020). In particular, among the different soft actuation technologies, soft pneumatic artificial muscles (PAMs) show promising potentials for industrial applications, due to their inherent customizability, robustness, low cost, and easy fabrication processes (Daerden and Lefeber, 2002; Bou Saba et al., 2019). Nevertheless, many of these PAMs suffer from a lack of sensing capabilities preventing the host system to achieve a real time monitoring of kinematics (i.e., deformation, force, etc.) and feedback control (Lu et al., 2020). In addition, due to the complexity in modeling the actuation dynamics exhibited by constitutive materials, the host systems are often built, based on an experimental study that implicitly accounts for the effects of uncertainties and uncontrollable variables in the morphology (Elgeneidy et al., 2018a; Tiziani and Hammond, 2020).

To address these issues, many PAMs have sensors embedded to measure either the actuation displacement or the exerted force for axial contraction (Tiziani et al., 2017; Wirekoh et al., 2019) in order to achieve a proprioceptive feedback control. Here, two main challenges are still open: 1) due to the non-linearity of the actuator and sensing constituent materials, as well as the considerable hysteresis between applied pressure and measured strain, the feedback control encounters inherent instabilities; 2) classic sensing solutions can encumber the overall design and affect the actuator deformation (and movement) and compliance (Tiziani and Hammond, 2020). For instance, the sensorized PAMs embedding rigid external sensors (i.e., linear encoder, load-cell) show a significant increase in the form factor and a reduced compliance (Erin et al., 2016). Indeed, a silicone elastomer generally has a Young’s modulus less than 10 MPa in the linear regime and high stretchability up to 500% (Case et al., 2015). For these reasons, a significant difference in mechanical properties (i.e., stiffness, deformability, etc.) between the actuator and sensor could strongly influence achievable movements. Therefore, soft sensors should be rigorously designed so that the sensorized PAMs achieve desired kinematic trajectories (Lu et al., 2020).

Several emerging approaches were explored for embedding soft sensing in PAMs and, more in general, in soft actuated structures. Among them, stretchable optical waveguides were widely exploited due to its high compliance. In particular, since these optical sensors detect a loss of optical power, the undesired mechanical failures (i.e., leakage, yield due to stress concentration, etc.) can be avoided. Thus, Zhao et al., presented the stretchable optical waveguides integrated into fiber-reinforced soft fingers for curvature, elongation, and force sensing (Zhao et al., 2016). Also, similar optical-based sensing mechanisms have been proposed in soft pneumatic actuators (Galloway et al., 2019; Jung et al., 2020) for proprioception.

Resistive sensing was also exploited for detecting the shape of soft actuators (Giffney et al., 2016; Elgeneidy et al., 2018b) by using conductive composites. While this solution could be useful due to a straightforward sensing mechanism, output characteristics are influenced by several external factors (i.e., temperature, humidity). In particular, most of them need a rigorous design to integrate with actuators; the intrinsic properties of soft materials must be carefully addressed because the performances of the sensor could present a large variability.

On the other hand, inductance-based sensors using a braided fiber mesh are robust in terms of less hysteresis, temperature or humidity drift, and limited bandwidths, rather geometry variations of the braided fiber (used as a reinforcement for the actuation) have an influence (Felt and Remy, 2014). Accordingly, by measuring the deformation of an elastomeric bladder, the smart braid can provide position feedback for the actuators. Moreover, since the length of the actuator is generally related to the actuator stiffness and the force output (blocking force), both displacement and force can be precisely predicted by measuring variations of the inductance and resistance (Felt et al., 2017). In particular, the stiffness due to the sensing element is symmetrically distributed along the longitudinal direction of the actuator. Indeed, such design principle allows for purely longitudinal trajectory of the actuator without undesired movements due to local and/or asymmetric stiffness.

In this work, we address the sensorization of a vacuum-powered PAM (instead of a positive pressure PAM). Given the abovementioned aspects, we select and investigate an inductive sensing solution for implementing a proprioceptive feedback control that can suppress uncertainties of the overall system, based on experimental driven approach. Vacuum-powered PAMs usually exhibit a large deformation with fast responses and offer implicitly fail-safe operations (i.e., avoiding delamination) (Robertson and Paik, 2017). In this case, the sensing can be challenging. The total stiffness due to the sensorization could be remarkably increased, while degrading the mechanical performances (i.e., achievable kinematic trajectories, blocking force, bandwidth, etc.) that the actuator should achieve. Indeed, the total axial stiffness of vacuum-powered PAMs strongly depends on Young’s modulus and its structural geometry, rather than imposed vacuum pressure. Thus, in general, it is more compliant and sensitive than those using positive pressure. The PAMs based on positive pressure undergoes a stiffening, as it becomes pressurized. Indeed, the buckling force or pressure (indicating the critical value for column squirm) is proportional to the axial stiffness, and to the imposed pressure, respectively. Hence, the actuator stiffness of positive pressure-based PAMs can be significantly enhanced. On the other hand, this cannot be done with vacuum-powered PAMs, since they generally are in bulk state upon the vacuum pressure; therefore, the overall stiffness of the actuator is strongly influenced by the constituent materials.

These facts imply that integrating mechanical components having different stiffness would influence the overall actuator stiffness, and prevent desired kinematic trajectories. For instance, a sensing element with relatively higher stiffness than the actuator could lead to the asymmetrical distribution of the overall architecture stiffness, resulting in local deformations (i.e., bending, twisting, etc.). Therefore, softer sensor having a compliance larger than the actuator's stiffness needs to be addressed. Moreover, from a material point of view, elastomeric transducer mechanisms cannot achieve high compressibility since their constitutive material has very limited compressibility (nearly incompressible).

Recently, porous materials have been exploited for both soft sensors and actuators due to their unique mechanical characteristics (i.e., high compressibility, elasticity, light weight, etc.). Indeed, many studies have explored resistive and/or inductive sensing solutions using porous materials (Liu et al., 2018; Luo et al., 2019; Wang et al., 2019). In particular, they can be used for either multimodal deformation sensing or tactile sensing by using several technologies [i.e., using specific electrodes configuration (Nakamaru et al., 2017), coating the conductive material (Nakayama et al., 2019), etc.]. Besides, due to the entangled air cells, the porous materials are quite useful to implement vacuum-powered PAMs. For instance, a vacuum-powered soft pneumatic actuator (V-SPA) (Robertson and Paik, 2017) was built by using a commercial foam and demonstrated in a hyper-redundant manipulator and locomotive robot. Our preliminary study on a bellow type PAM using a polyurethane foam demonstrated promising mechanical performances (i.e., deformation ratio of 52% and blocking force of 3 kgf) (Joe et al., 2021). For both cases, the foam was commonly used for a core architecture to obtain its high compressibility. It was encased by an elastomeric skin to allow the flows to be pressurized and/or depressurized.

In this work, we propose an inductive-based soft deformation sensor embedded in our vacuum-driven, bellow-type PAMs. First of all, the working principle of this novel solution is introduced for ultralight and highly compressible actuators; then, the characterization of the sensorized muscle is discussed. Finally, closed loop control system using the deformation sensing information is demonstrated, showing that the sensorized muscle can achieve accurate position control, as well as detect the disturbance and compensate it.

## 2 Materials and Methods

### 2.1 Conceptual Design

**FIGURE 1:**
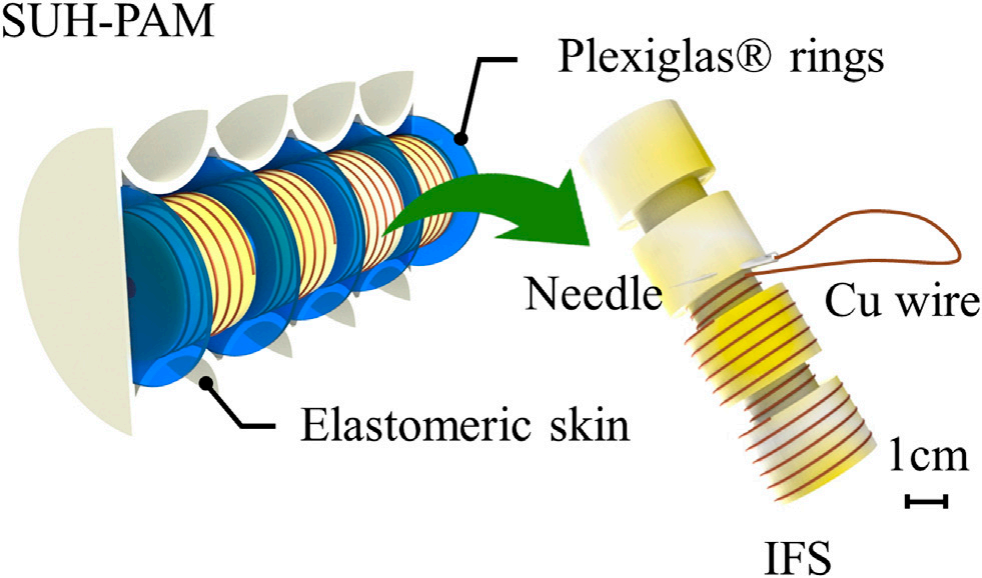
Concept design of Sensorized Ultralight Hybrid Pneumatic Artificial Muscle (SUH-PAM), consisting of elastomeric skin, plexiglass rings, and Inductive Foam Sensor (IFS).

The SUH-PAM consists of Plexiglas^®^ rings, IFS and elastomeric bellow skin, as shown in Figure 1. In our preliminary study (Joe et al., 2021), the optimal dimension and configuration of elastomeric bellow skin, having four convolutions with 1 mm thickness, was obtained by means of FEM modeling. The Plexiglas^®^ rings integrated at each convolution reinforce the axial stiffness, while avoiding undesired structural deformations (i.e., column squirm and/or buckling). The polyurethane foam composed of entangled air cells enables highly deformable and ultralight. In particular, it can be employed in PAMs design by encasing them with an elastomeric skin. Based on such mechanical structure, the IFS embedding the helix-shaped conductive wire can provide information about its biaxial deformations (both contraction and extension).

### 2.2 IFS Operation Principle

The core three-dimensional sensitive soft structure consists of a cylindrical open-cell foam with a Cu coil shaped as a helix along its lateral surface. A helical coil is structurally compressible due to the structure (like a compression spring), which makes it ideal for embedding into the UH-PAM without affecting the actuator’s characteristics. The inductance of the helical coil made of the conductive wire is determined by the pitches of the helix. When the helical coil is compressed, the coil loops move closer to each other (smaller pitch), which gives higher magnetic field coupling, thereby higher self-inductance of the coil. From the Wheeler’s formula (Wheeler, 1928), the correlation between the inductance and the number of turns with a height can be written as:

$$L_{0}=\frac{N^{2}D^{2}}{450D+1,000h_{0}},$$ (1)

where *N* and *D* refer to the number and diameter of coils, respectively. *h* is the height of helix. This formula is correct to within 1% for coils when the height is 0.4 times greater than diameter (*h > 0.4·D*). In addition, these parameters should be millimeter (mm) scale. If *N* and *D* are constants, the only *h* is then variable, and Eq. 1 can be written as:

$$L=\frac{N^{2}D^{2}}{1,000}\cdot \frac{1}{0.45D+h}.$$ (2)

Hence, once the external force or displacement applies at a foam, the height of foam (*h*_*0*_) decreases along the longitudinal direction, the inductance increases. Therefore, the normalized inductance variation can be expressed as a function of the compression strain (*ε = ∆h/h*_*0*_) and the coil’s geometry factor (*k*):

$$\frac{\Delta L}{L_{0}}=\frac{1}{1-k\cdot \epsilon }-1=\frac{-\Delta h}{0.45D+h_{0}},$$ (3)

where *k* is a coefficient of the geometry of the coil, which can be written as:

$$k=\frac{h_{0}}{0.45D+h_{0}}.$$ (4)

Equations 1–4 are valid only with some assumptions. In particular, in addition to neglecting the non-linear foam material properties (i.e., viscoelasticity, porosity, etc.), two important conditions are that the coil-foam relative position is not altered upon foam deformation, and the foam is uniformly deformed along with its longitudinal axis.

### 2.3 Fabrication

Part A and B of Dragon Skin 30 (Smooth-on Inc., Easton, PA, United States) were mixed at a weight ratio of 1:1 and degassed by a vacuum chamber. The molds for casting the two halves of elastomeric bellow skins were made by 3D printing (ProJet MJP 3600, 3D Systems, South Carolina, United States). Then, the mixed silicone was poured into the molds, respectively, and both skins were cured for 15 h. A commercial open-cell foam made of polyurethane (8643K549, McMaster-CARR, Chicago, United States) was used for the cylindrical segments. Each segment consists of four cylinders (20 mm diameter and 12.4 mm length) and three bridge cylinders (11 mm diameter and 6.2 mm length). Then, the foam structure was built by bonding all segments with silicon adhesive (SIL-Poxy^®^ adhesive, Smooth-On Inc.) The Cu wire (BLOCK, FA-Nt.: 431101) with a diameter of 0.2 mm was manually sewed around the foam structure in a helix shape using a needle. The rings (28.5 mm diameter) were derived from a Plexiglas^®^ layer of 0.8 mm thickness and patterned with the 2D same laser cutter (Versa LASER VLS 3.5). The rigid mask and the sensorized foam structure were integrated with elastomeric skins that detached from the molds. Here, since the foam could absorb the liquid silicone, all components were bonded to the foams by brushing the same silicone (Dragon Skin 30). After curing the silicone, both halves of bellow skins were bonded together [see Supplementary Figure S1 in Supplemental Material (SM)].

### 2.4 Experimental Setup

#### 2.4.1 Compression Tests of Foam Samples With Embedded Helical Coil

**FIGURE 2:**
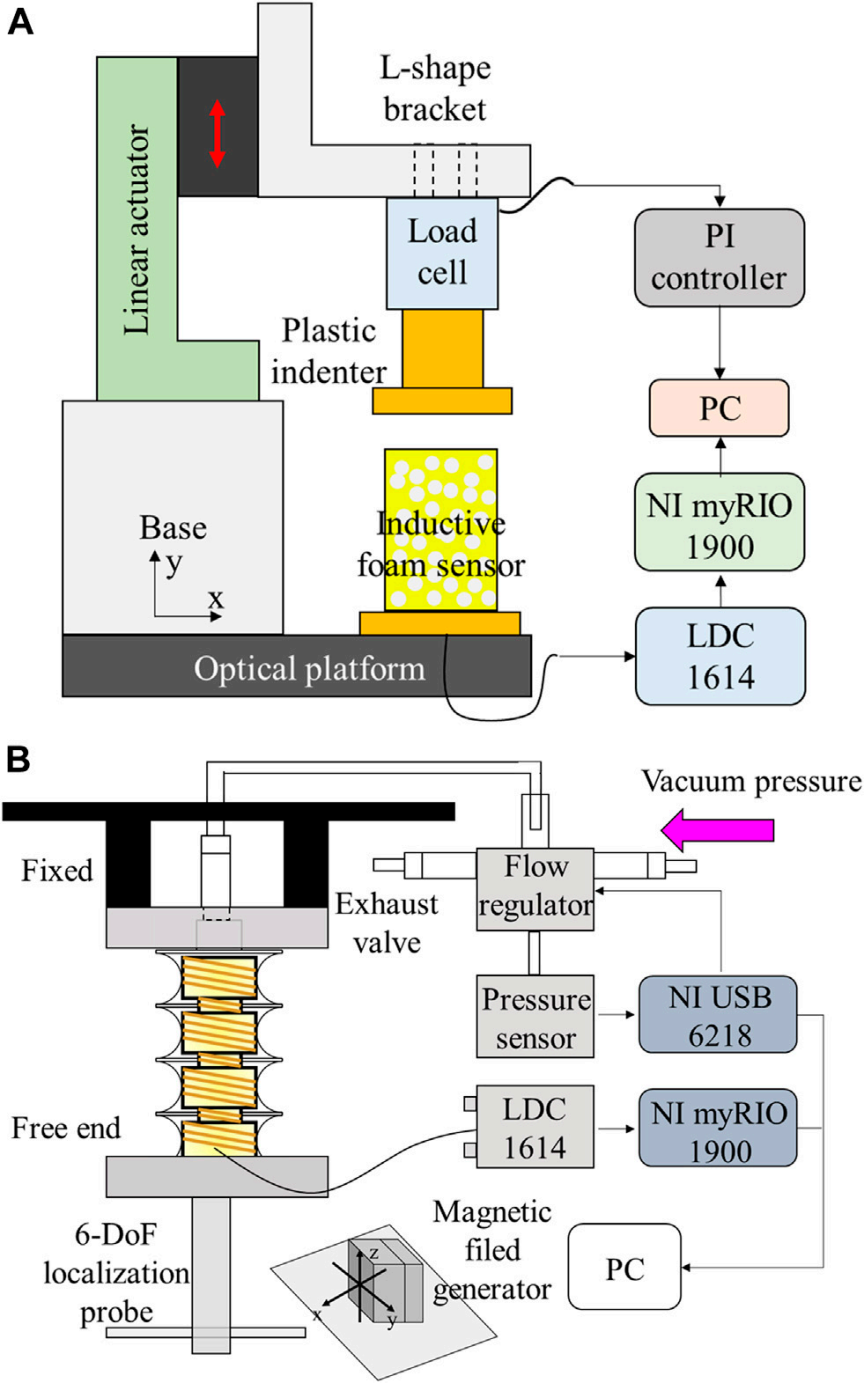
Experimental setups. **(A)** Compression test platform for the foam samples with embedded helical coil. **(B)** SUH-PAM characterization platform.

The displacement was induced by means of a motorized linear actuator (M-111.1DG, Physik Instrument, Germany). Along the longitudinal direction, the system could be controlled at the micrometer scale and generate a deformation of up to 15 mm, enabling the strain 59% with respect to the initial height of cylindrical foam. A single-axis load cell (FSH00103, FUTEK, CA, United States) was interfaced with a plastic indenter (23 mm of diameter) and used to measure the compression force applied on the foam. A fully integrated digital inductance converter evaluation board (LDC1614 EVM, Texas Instruments, United States) was exploited to measure the inductance based on the oscillation frequency. A LabVIEW program was developed for data acquisition and analysis through a NI-myRIO 1900 (National Instruments, TX, United States), as shown in Figure 2A.

#### 2.4.2 Characterization Platform of SUH-PAM

The output pressure of the vacuum flow regulator (ITV0090, SMC corporation) was set to the desired pressure. The flow regulator can control the vacuum pressure, ranging from 0 to −100 kPa, which had a built-in pressure feedback system. The pressure control of the vacuum flow regulator was realized by means of the analog input signal. The input signal is proportional to the output pressure in case flow fluctuation is absent. The vacuum pump (Diaphragm Vacuum Pumps and Compressor, N022 AN.18, KNF LAB, Balterswil, Switzerland) generates a maximum vacuum of 100 mbar. Acquisition and generation of an analog signal for the pressure control and of the digital signal for the foam sensor were implemented by DAQ6218 and myRio 1900 (National Instruments, TX, United States), respectively. To investigate kinematic responses (i.e., displacement, angle, workspace,etc.), AURORA electromagnetic tracking system (AURORA^®^ EM) was exploited with 6-DoFs a localization probe (Northern Digital Inc.), as shown in Figure 2B. By generating an electromagnetic field where the sensors are tracked, the electromagnetic tracking system provides real-time tracking at the working frequency of 800 Hz and a measurement rate of 40 Hz, respectively (Aurora, 2021). Meanwhile, the inductive sensor is worked at much higher level, ranging from 5 to 10 MHz. Herein, the measurement systems only detect the magnetic field signal at the exact frequency, and thus they do not affect each other. Indeed, such feature has been widely applied into practical applications. For instance, a radio using different electromagnetic frequency spectrum does not interfere each other, and it allows for multiple channels at different frequencies and picking up one of them without interference.

## 3 Results and Discussion

### 3.1 Sensing Characterization

**FIGURE 3:**
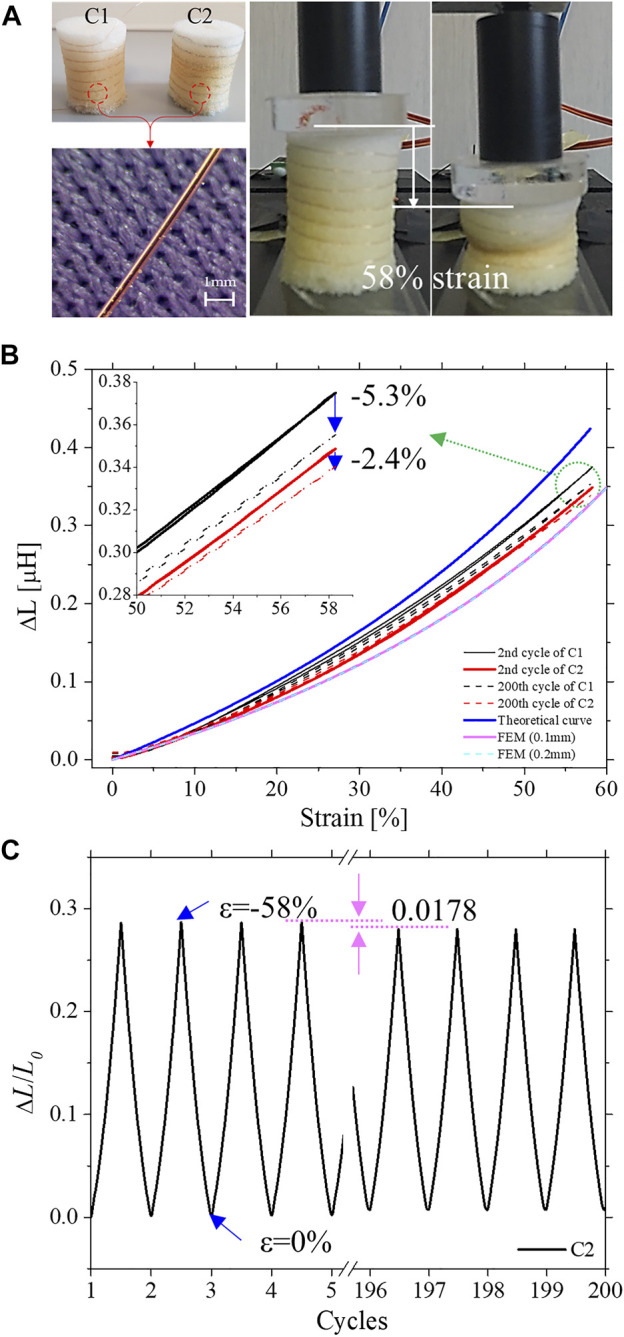
IFS response. **(A)** Fabricated SFIS that either wrapped (C1) or sewed (C2) a Cu wire along with a helix. For both C1 and C2 the following materials were used: Cu wire (BLOCK, FA-Nt.:431101, diameter 100 μm) of same length and helix turns (20 cm and 7 turns); cylindrical open-cell foams (diameter: 19 mm, length 25.5 mm). **(B)** Strain-normalized inductance curves of 2nd and 200th cycle for each case, with theoretical and FEM results. **(C)** The plotted normalized inductance-cycles relationship of C2 with the sensitivity drift due to the permanent crushing of its cellular structure.

A compression test platform was used to characterize the proposed sensing solution. As shown in Figure 3A, the foam sensor using a Cu wire of 100 µm is deformed by 58% contraction, while the inductance is reduced by 1.008 µH. To identify the inductance variation according to the diameter of Cu wire, simulations using the Cu wire of 200 µm and of 100 µm were performed by means of AC/DC module of COMSOL, and details of the COMSOL simulation results are presented in Figure 3B *.* For different diameters of Cu wire, a significant difference in the inductance variation was not observed. Therefore, it can be concluded that the diameter of Cu wire does not affect the performance of the IFS. However, since the foam's air cells absorb the used material and cause unexpected behaviors when pressure, displacement, or force are applied, direct bonding of Cu wires and foam by silicone, rubber, or glue should be avoided in order to successfully fabricate the foam sensor. Therefore, two possible fabrication solutions were tested: The Cu wire was either wrapped, for Case 1 (C1), or wrapped and sewed, for Case 2 (C2), around the foam cylinder, respectively (in C1 only the extremities of the wire were locally bonded by glue to the foam).

The dynamic characteristics of C1 and C2 prototypes (obtained by means of the developed instruments described in the experimental setups section) are shown in Figure 3B. First, we analyzed the mechanical behavior of C1 and C2 sensors. In both cases, a highly viscoelastic behavior can be observed, as already investigated in our previous studies (Wang et al., 2019; Joe et al., 2020; Totaro et al., 2020), with a relevant hysteresis. Moreover, both samples were tested with 200 loading/unloading cycles for 8 h, showing the variations of mechanical characteristics (as displayed in the dashed curves of Figure 3B and in normalized inductance versus cycles of Figure 3C). Indeed, repeated compression or impact loading can cause a loss of elasticity in the foam, and this behavior is commonly observed in the literature (Ramirez et al., 2018). Furthermore, the mechanical hysteresis is varying, due to the permanent crushing of its cellular structure. In principle, this behavior could strongly affect the performance and the reliability of sensing devices based on such commercial foams. These limitations can be overcome by developing custom-made foam structures, with finely tuned porosity and mechanical properties of the constituent material (Goswami et al., 2019).

However, here we demonstrate that the proposed sensing solution can allow the use of such materials in highly sensitive and low costly soft sensors, and hysteresis-free response. Indeed, in Figure 3B, the inductance variation (*ΔL*) versus strain characteristics is shown for both C1 (red-lines) and C2 (black-lines) sensors, compared with the theoretical (blue line) prediction of Eq. 1 and of FEM, respectively. In the 2nd cycle, the average error rates of C1 and C2 with respect to theoretical values (and FEM) were 12.58% (FEM: 22.8%), and 20.01% (FEM: 9.42%), respectively. However, after the 200th cycle, the error rates of C1 and C2 were increased up to 19.3% (FEM: 21.55%) and 21% (FEM: 19.12%), respectively. In the case of C1, 6.7% of the error rate increased owing to the sensitivity drift. On the other hand, the error rate of C2 increased only of 1%.

From a static point of view, both curves show very similar behavior. On the other hand, dynamic characteristics show that the sensor characteristics showed a sensitivity drift for C1 and C2 of only 5.4 and 2.4%, respectively, after 200 loading/unloading cycles. The relatively worse results observed in C1 are mainly due to the probable undesired slippage between the Cu wires and the foam modules. On the other hand, C2 showed relatively high reliability with less sensitivity drift. For this reason, C2 was identified as a better fabrication solution to implement the helical coil sensor into the SUH-PAM.

### 3.2 SUH-PAM Static Behavior

**FIGURE 4:**
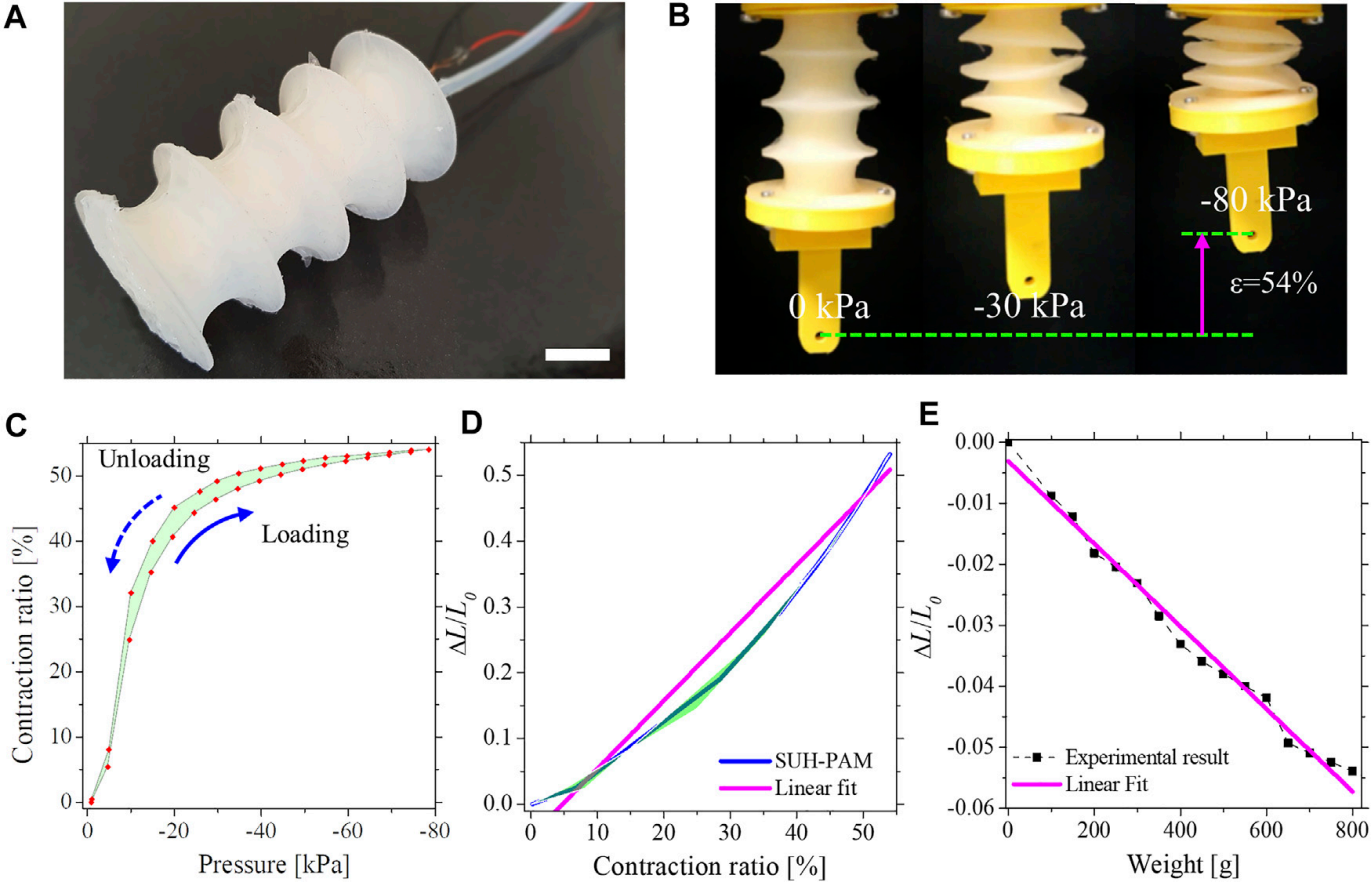
SUH-PAM static characteristics. **(A)** SUH-PAM with IFS integrated (scale bar corresponds to 10 mm). **(B)** Photographs of the SUH- PAM at the vacuum pressures ranging from 0 to −80 kPa (Max contraction ratio = 54%). **(C)** Contraction ratio-pressure curve. **(D)** Normalized inductance-contraction ratio, with a linearly interpolated line (pink). **(E)** Relation of weights and normalized inductances, and the linearly interpolated normalized inductance line (pink).

The SUH-PAM with integrated IFS is shown in Figure 4A. Following the fabrication of the SUH-PAM, silicone tubing was inserted at one terminal of the actuator to enable depressurization, and the Cu wire free end was connected to the inductance measurement board (LDC 1614 EVM, Texas Instruments, United States). By exploiting the characterization platform, the SUH-PAM can be fully contracted up to 54% upon a vacuum pressure of −80 kPa, as shown in Figure 4B. The hysteresis between the contraction ratio and the pressure was 8.39%. These mechanical characteristics are quite similar to the UH-PAM (Joe et al., 2021), indicating that the embedded Cu wire does not affect the mechanical behaviors of the PAM itself. Meanwhile, the hysteresis between the inductance and the contraction ratio was 5.35%, and the average error rate was only 1.85%. In particular, the contraction ratio versus pressure curve shows a non-linear behavior, while the normalized inductance variation versus contraction ratio curve shows a linear behavior (*R* ^2^ = 0.974), as from Figures 4C,D. The sensitivity of SUH-PAM was identified as 0.0103/1% (Normalized inductance/Contraction ratio). The inductive sensing results much more reliable and precise than pressure sensing, which allows the host system to perform a proprioceptive feedback control by measuring the variation of inductance due to deformations.

In addition to longitudinal compression, the elongation of the SUH-PAM owing to an external weight was investigated by imposing additional loads ranging from 0 to 800 g. To correlate the weight (*w*) with the normalized inductance variation (*∆L/L0*), a Least Square Method (LSM) was exploited. The relation of the weight versus the normalized inductance shows not only a high sensitivity of 14566.71·(*∆L/L0*)-39.16, but also good linearity (*R*2 = 0.986) (Figure 4E). The inductance variations concerning the two weights (234 and 522 g) were acquired as −0.0197 and −0.0412, respectively. With the normalized inductance variation of −0.0197, the LSM function approximated the weight of 247.1 g. Similarly, with a variation of −0.0412, the expected weight was 561.79 g. Indeed, the error rates were 5.6 and 7.6%, respectively.

### 3.3 SUH-PAM Dynamic Behavior

A sinusoidal input pressure with an amplitude of −80 kPa was applied at varying frequencies, ranging from 0.025 to 0.2 Hz, in order to identify the dynamic characteristics of SUH-PAM (i.e., bandwidth, deformation response, etc.) with respect to full contraction of 54% and a null load. The resulting sinusoidal inductance variation that corresponds to the SUH-PAM deformation was recorded, as an output. The magnitude corresponds to a logarithm of the response ratio between the reference inductance value (at 54% contraction with no load) and the actual inductance value at different frequencies.

**FIGURE 5:**
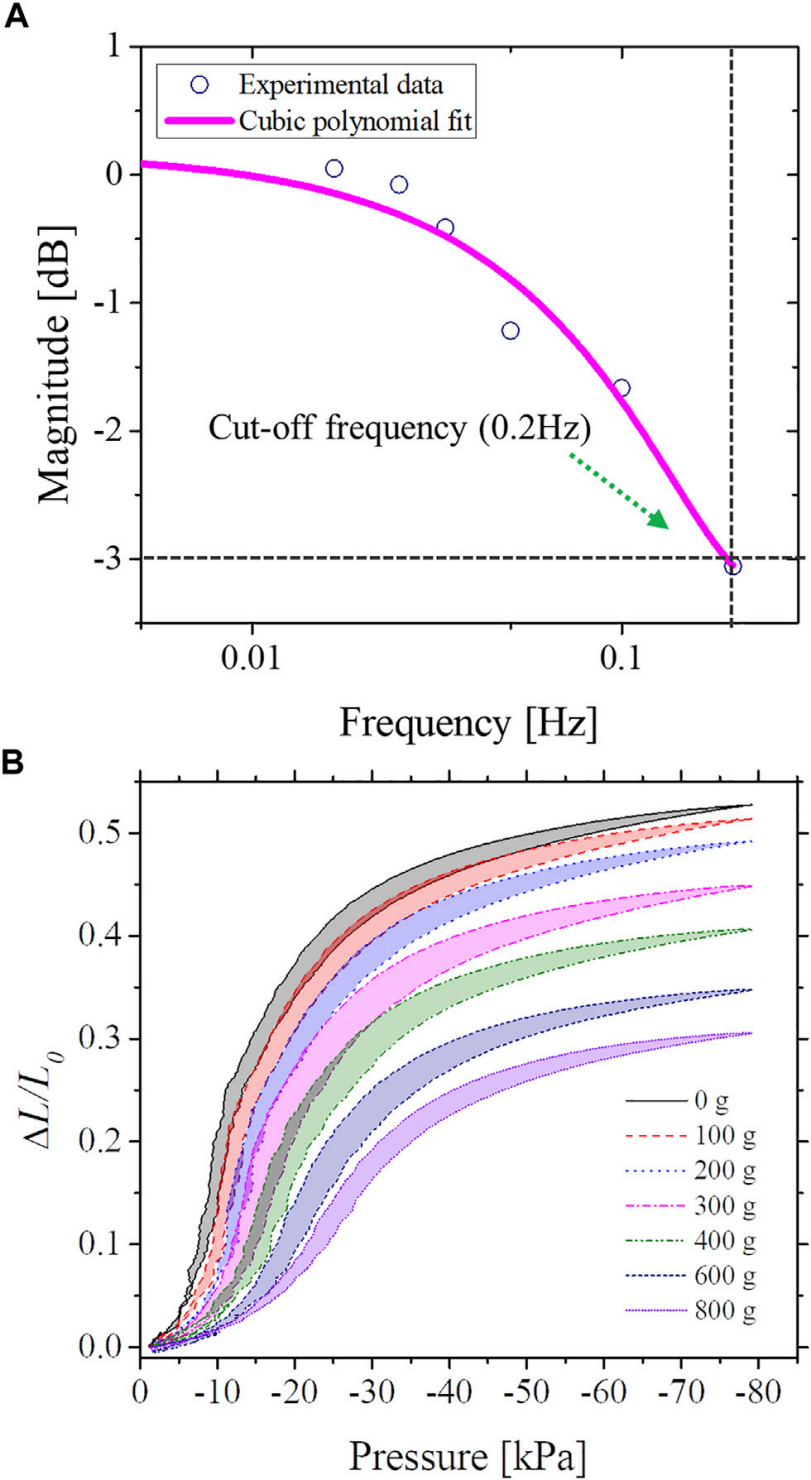
SUH-PAM dynamic characteristics. **(A)** Frequency response of SUH-PAM (cut-off frequency of 0.2 Hz). **(B)** Dynamic responses (i.e., pressure and normalized inductance) for payloads from 0 to 800 g.

As shown in Figure 5A, the obtained magnitude as a function of the frequency was interpolated by means of a cubic polynomial fitting (Robertson et al., 2017). The response was gradually attenuated, and the −3 dB response was found at 0.24 Hz, which corresponds to the cut-off frequency. Therefore, the operational frequency range of the SUH-PAM can be ensured up to 0.2 Hz. This value is mainly limited by the used flow regulator. To further identify the dynamic characteristics of SUH-PAM, a square wave and random step load, with respect to a full span from 0 to 8 V (−80 kPa) were used (further details reported in Step responses in SM section 1).

As shown in Figure 5B, we performed an experiment by imposing different weights from 0 to 800 g and monitored the dynamic response using a sinusoidal wave. Interestingly, the heavier weight, the less variation of inductance was observed. In case of null weight and no load, a full span of the normalized inductance was 0.52, while it was decreased down to 0.3 when the weight of 800 g was imposed. For all cases, the hysteresis and non-linearity of SUH-PAM were observed.

### 3.4 Feedback Control

Despite the SUH-PAM showed promising mechanical characteristics, realizing that proprioceptive feedback control is challenging due to its relevant hysteresis and non-linearity, between sensing output and control parameters. Indeed, the characterization results of SUH-PAM imply that the system can undergo uncertainties and/or instabilities. Such difficulties can be addressed by a robust and effective feedback control so that the proprioceptive soft actuator could saturate up to the desired state. To this aim, we selected unmodeled control method by combining a sliding mode control (SMC) and a proportional gain (*K*_*p*_). This control strategy is useful due to its simplicity and promising capabilities (i.e., high precision, fast response, robustness, etc.).

In working principles, the SMC allows the system to converge towards a selected surface and remain in its state (Edwards and Spurgeon, 1998; Ghabi et al., 2018). Generally, the switching constant should be large enough to suppress all matching uncertainties and unexpectable system of dynamics (Furat and Eker, 2012). Due to a robustness of the SMC, proprioceptive and/or exteroceptive soft actuators have been widely employing for their feedback control (Luo et al., 2017; Wirekoh et al., 2019). However, due to a large switching constant, the SMC could suffer from the phenomenon of finite-amplitude oscillations, also called chattering (Guldner and Utkin, 2000).

Similarly, we observed the chattering during a position control of SUH-PAM, as addressed in sliding mode control in SM section 2. In particular, if the switching constant is sufficiently large (see Supplementary Figures S3B,C in SM), the chattering has then occurred with a huge vibration, while the response against the disturbance becomes fast. To reduce the chattering while achieving benefits of SMC control, adaptable proportional sliding mode control (P-SMC) is employed, as similarly reported in (Banrejee and Nigam, 2011; Komijani et al., 2020). Then, we focused on compromising control parameters (i.e., *K*_*P*_ and *K*_*s*_) allowing for robustness against disturbance and for reducing the chattering.

**FIGURE 6:**
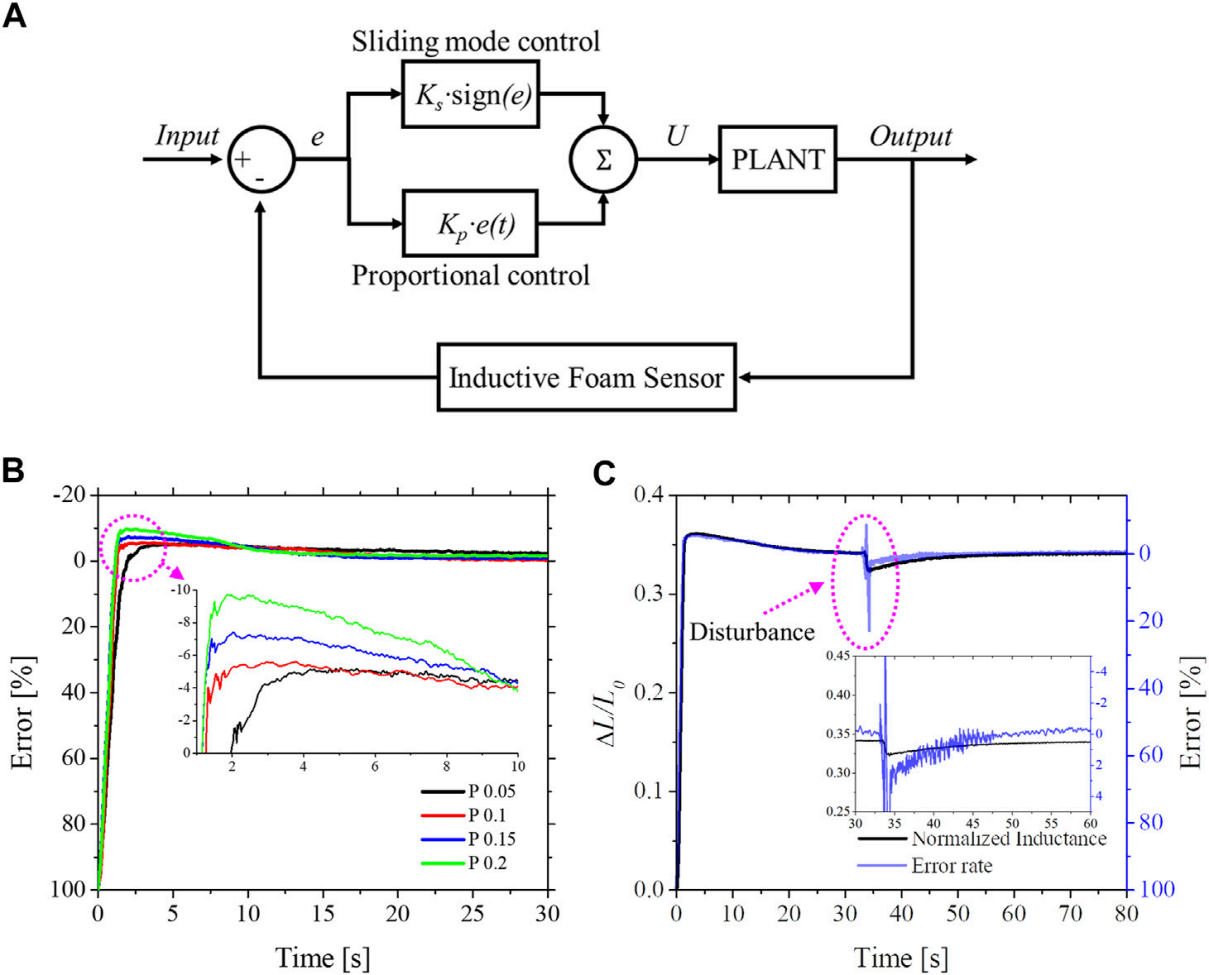
SUH-PAM with feedback control system. **(A)** Block diagram of the Proportional Sliding Mode Control (P-SMC). **(B)** The error rate according to P gains. **(C)** The system response of P-SMC during position control, showing adaptability against the disturbance.

As shown in Figure 6A, the controller implements both a proportional gain and a proportional scheme that alternated between a large and small gain. The modified control law is as follows:

$$\begin{array}{l} U=U_{P}+U_{s}=K_{P}\cdot e\left(t\right)+K_{s}\cdot \text{sign}\left(e\right)\\ \mathrm{With:}\\ \text{sign}\left(e\right)=\{\begin{array}{c} 1,e>0\\ 0,e=0\\ -1,e<0 \end{array}, \end{array}$$ (5)

where *e* indicates an error rate between the current state and desired state (i.e., setpoint), and the *K*_*p*_ is a proportional gain. Due to the switching constant (*K*_*s*_), the system is able to robust against the disturbance and uncertainties of the model. Here, by adding a proportional gain, the system can further achieve adaptability and fast response to saturate its desired state. To evaluate the performance of the presented feedback system, several control parameters (i.e., rise, settling times, and overshoot) were used. Then, we focused on the response time of the system concerning the disturbance and obtained an optimal proportional gain that minimizes the overshooting and undershooting. As shown in Figure 6B and Table 1, with *K*_*p*_ of 0.1 and *K*_*s*_ of 0.0001, the response was the fastest, and the rise time and settling time were 0.88 and 6.82 s, respectively. Here, a threshold of 5% for defining settling time was identified.

**TABLE 1:** Characteristics of P-SMC for a desired displacement.

*K*_*p*_ gain	0.05	0.1	0.15	0.2
Rise time [s]	1.244	0.877	0.821	0.819
Settling time [s]	7.435	6.822	9.182	8.943
Peak time [s]	5.05	3.775	2.025	1.875

*K_*s*_ is fixed to 0.0001.

Similar to the general characteristics of the PID control scheme, the greater P gain, the faster rise time. On the other hand, both the increased settling time and overshooting were observed (Figure 6B). A load variation, one of disturbance classes from outside of control loop (i.e., setpoint changes, load variation, and noise), was employed to generate the repeatable disturbance and was imposed at the free-end of SUH-PAM.

For the disturbance, the SUH-PAM responded with a fast saturation time of 8 s, as shown in Figure 6C. Conclusively, the P-SMC was capable of SUH-PAM displacement control in mm scale with a steady-state root-mean-square error (RMSE) of 0.925 mm and enabled the response against the disturbance to saturate at the desired state within a relatively fast time.

## 4 Conclusion

The main results of this work concern the design and development of the sensorized PAM (SUH-PAM) with a simple and low-cost fabrication method (less than 1 USD, see Supplementary Table S1 in SM), with the implementation of a feedback control system that allows SUH-PAM to be robust against disturbances.

From a mechanical and sensing point of view, the compression test of the IFS highlighted that C2 (sewed Cu wire) shows higher reliability and precision than C1 (wrapped Cu wire). Moreover, the IFS could measure the true strain of the SUH-PAM. Indeed, the static characterization results of SUH-PAM demonstrated that the inductive sensing has a hysteresis of 5.35% and a sensitivity of 0.01031% for the deformation. Herein, the hysteresis is mainly due to the SUH-PAM components (i.e., foam, elastomeric skin, and rigid rings). The above findings on the IFS play a crucial role in implementing proprioceptive sensors capable of highly compressible with high sensitivity. Thus, it is envisioned that the presented sensing solution can be expanded for other soft robotic applications.

Meanwhile, the SUH-PAM has a linear behavior of the imposed weight versus the variation of inductance. Then, in principle, a control system based on the imposed weight could be implemented. The SUH-PAM can also lift the weight up to 800 g, corresponding to 38 times of its own weight (21 g). The characterization results illustrated that the SUH-PAM has a strong potential to detect deformations due to a negative input pressure and external load, while enabling real-time sensory feedback. From the material point of view, it is remarkable that the IFS allows for biaxial deformations while ensuring a high sensitivity. Moreover, in terms of exteroception concerning the load variation, the IFS has shown a strong potential, allowing for developing a multimodal sensing solution. Finally, a proprioceptive feedback control was demonstrated by means of P-SMC, which provides robustness against disturbances and suppresses instabilities and uncertainties that the host system could have.

The results of this work are useful to highlight that with a simple and low-cost approach a smart vacuum-based PAM, with proprioceptive feedback control, can be achieved. However, the presented coil-based inductive sensing solution could suffer from the following limitations. First, from a sensing point of view, it could limit its practical uses only for soft actuators and robots made of silicone or plastic body material, since the SUH-PAM could not interact with objects made of conductive and/or ferromagnetic materials. Moreover, to aim for high sensing performances (i.e., accuracy, sensitivity, etc.), scaling down the overall dimensions of the IFS (e.g., reducing diameter or height) is necessary. However, difficulties in fabrication and integration could limit its overall dimensions down to mm scale. Secondly, the limits regard the fabrication method used that relies on integrating a Cu wire in completely soft-porous polyurethane foam, that affects the reliability of prototypes. Indeed, we observed a slight difference in the inductance among different SUH-PAM prototypes, which implies the need of characterizing and calibrating each prototype.

However, these preliminary results can provide inspiration for new material science solutions that can address the integration of a highly conductive material in a porous matrix. Hence, future works will focus on the investigation of novel porous materials (passive and conductive) by exploiting additive fabrication protocols that can improve mechanical robustness and ensure reproductivity and repeatability.

## Data Availability

The original contributions presented in the study are included in the article/Supplementary Material, further inquiries can be directed to the corresponding authors.
