# Supplementary material for: Sensing Deformation in Vacuum Driven Foam-Based Actuator via Inductive Method
Source: Front Robot AI. 2021 Dec 14;8:742885. doi: 10.3389/frobt.2021.742885 (PMC10262191; doi:10.3389/frobt.2021.742885)
Supplement: Supplementary file 1 [file DataSheet1.pdf]

Supplementary Material

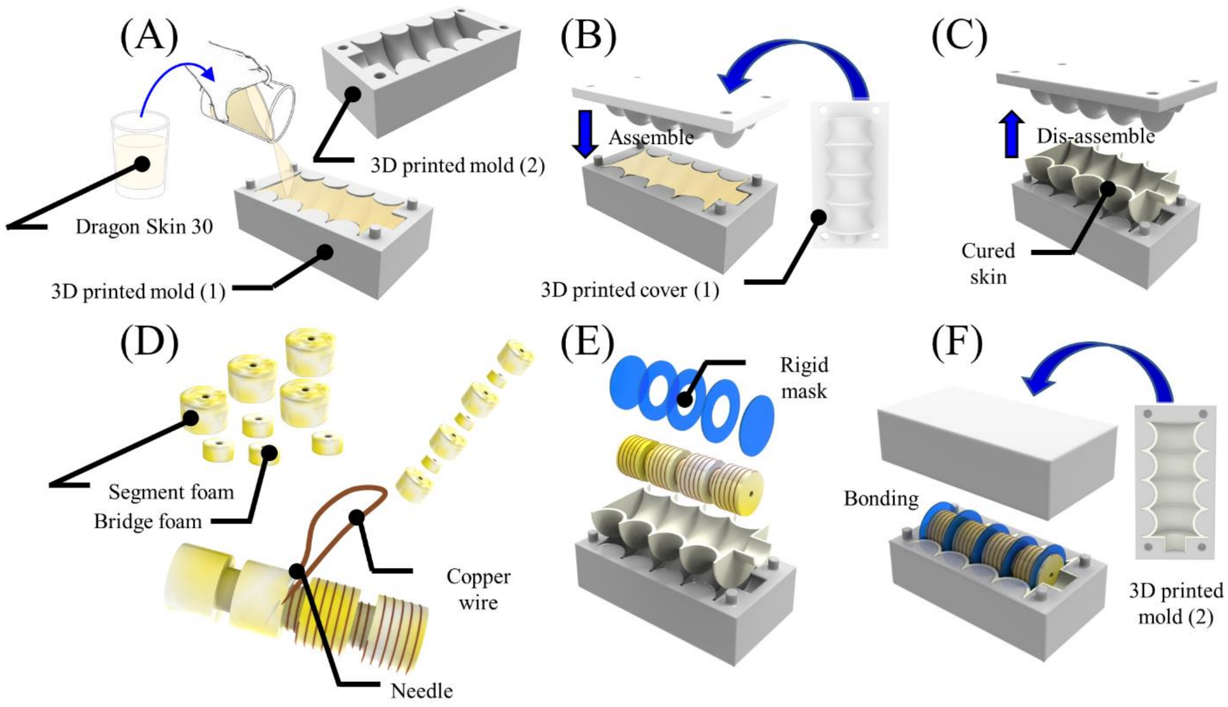

Figure S1. SUH-PAM fabrication

1 Step responses

The dynamic characteristics of SUH-PAM were evaluated by using a square wave and random step load, with respect to a full span (from 0 to 8 V (-80 kPa)). As shown Figure S2A, there was no overshooting or undershooting, with respect to a step response. The embedded inductive foam sensor was compliant well concerning the deformation of SUH-PAM. The range of the inductance variation was from 0 to 52 %. However, The SUH-PAM showed two different rising times: The rising times of the contraction and releasing phases were 1.375s and 3.775s, respectively, with respect to the full span of -80 kPa. Since the flow regulator controls both pressures and flows supplied from the vacuum pump, the compressed internal volume of SUH-PAM can be passively recovered by releasing atmospheric pressure through a breath hole. For this reason, the releasing SUH-PAM needs more extended time to saturate its desired trajectory.

Meanwhile, the responses (i.e., strain and inductance) for the random step load were quite well matched with input and vacuum pressure, as shown in Figure S2B.

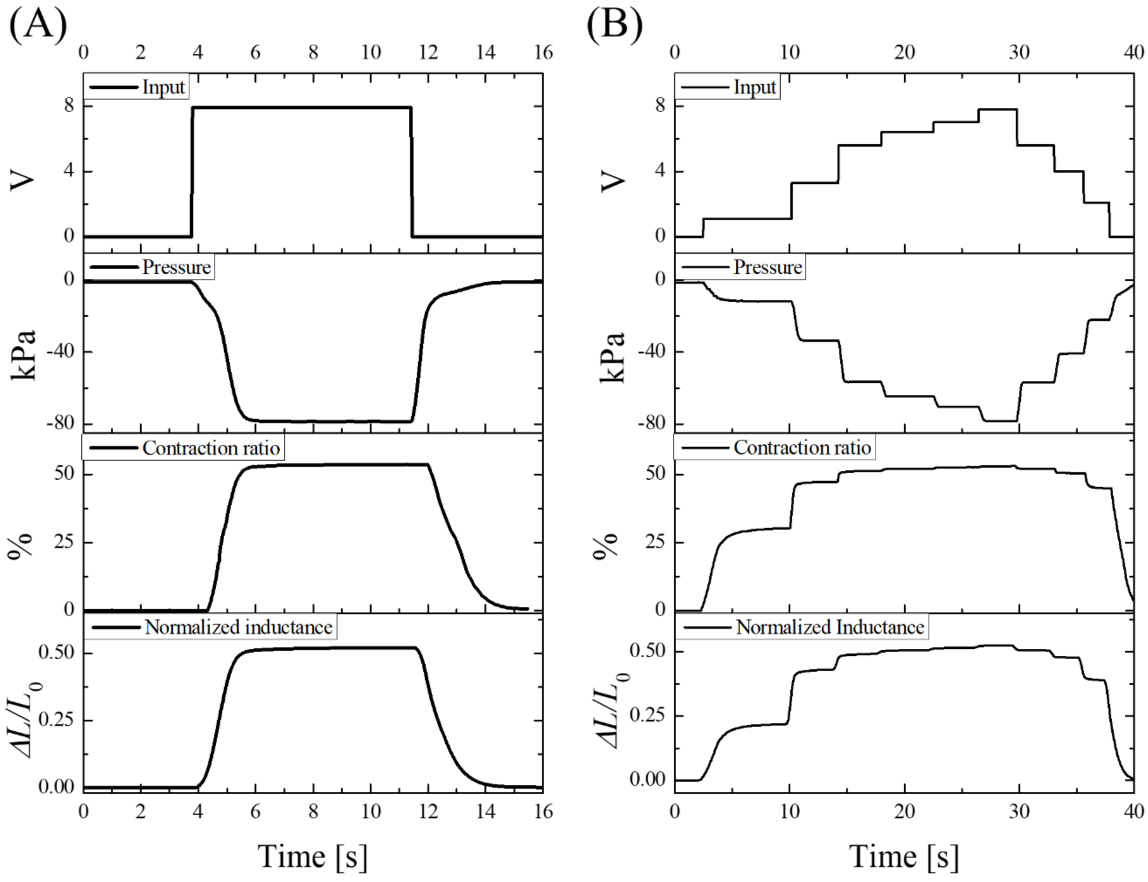

Figure S2. (A) Step response for a full span ranging from 0 to -80 kPa), (B) Dynamic responses for the arbitrary input voltages.

Table S1. Cost of materials used in the fabrication of the SUH-PAM.

Properties	Price	Used for SUH-PAM	SUH-PAM
Silicone skin	3.25 [USD/*l*]	0.016 [*l*]	0.053
Plexiglass	107.64 [USD/m^2]	0.002306 [m^2]	0.25
Silicone tube	0.17 [USD/m]	0.25 [m]	0.04
Copper wire	0.0417 [USD/m]	1 [m]	0.04
Foam	0.0047 [USD/cm^3]	16.921 [cm^3]	0.08
Total cost			0.46 USD

2 Sliding Mode Control (SMC)

The fundamental concept of SMC was derived from variable structure control. Due to its robustness and simplicity, the SMC allows the system to converge towards a selected surface and then to remain its state, despite of uncertainties and disturbance. The control law is as following;

$$\boldsymbol{U = K_s sign\ (e)}$$

With:

$$\boldsymbol{Sign(e)} = \begin{cases} \boldsymbol{1\ , e > 0} \\ \boldsymbol{0\ ,\ \ e = 0} \\ \boldsymbol{-1\ , e < 0} \end{cases} \tag{1}$$

where sign is the sign function and K_s is a positive (switching) constant that represent the discontinuous control gain. The controller implements a proportional scheme that alternated between a large and small gain, and the switching constant should be large enough to suppress all matching uncertainties and unpredictable system of dynamics. With a proper switching constant, the system is able to robust against the disturbance and model's uncertainties. Due to these, the systems inherent in instabilities (i.e., delay, hysteresis, non-linearity, etc) improve the accuracy and stability between the current state and desired state. When K_s was 0.01 or 0.001, the rise times were 1.7s and 1.8s, respectively, as summarized in Table S2. However, the responses showed the chattering as shown in Figure S3B and C. On the other hand, with the *Ks* of 0.0001, the rise time and settling time were measured to 0.8662 s and 1.448 s, respectively. In particular, the reduced chattering was observed, while the saturation took a long time to compensate the disturbance, as shown in Figure S3D.

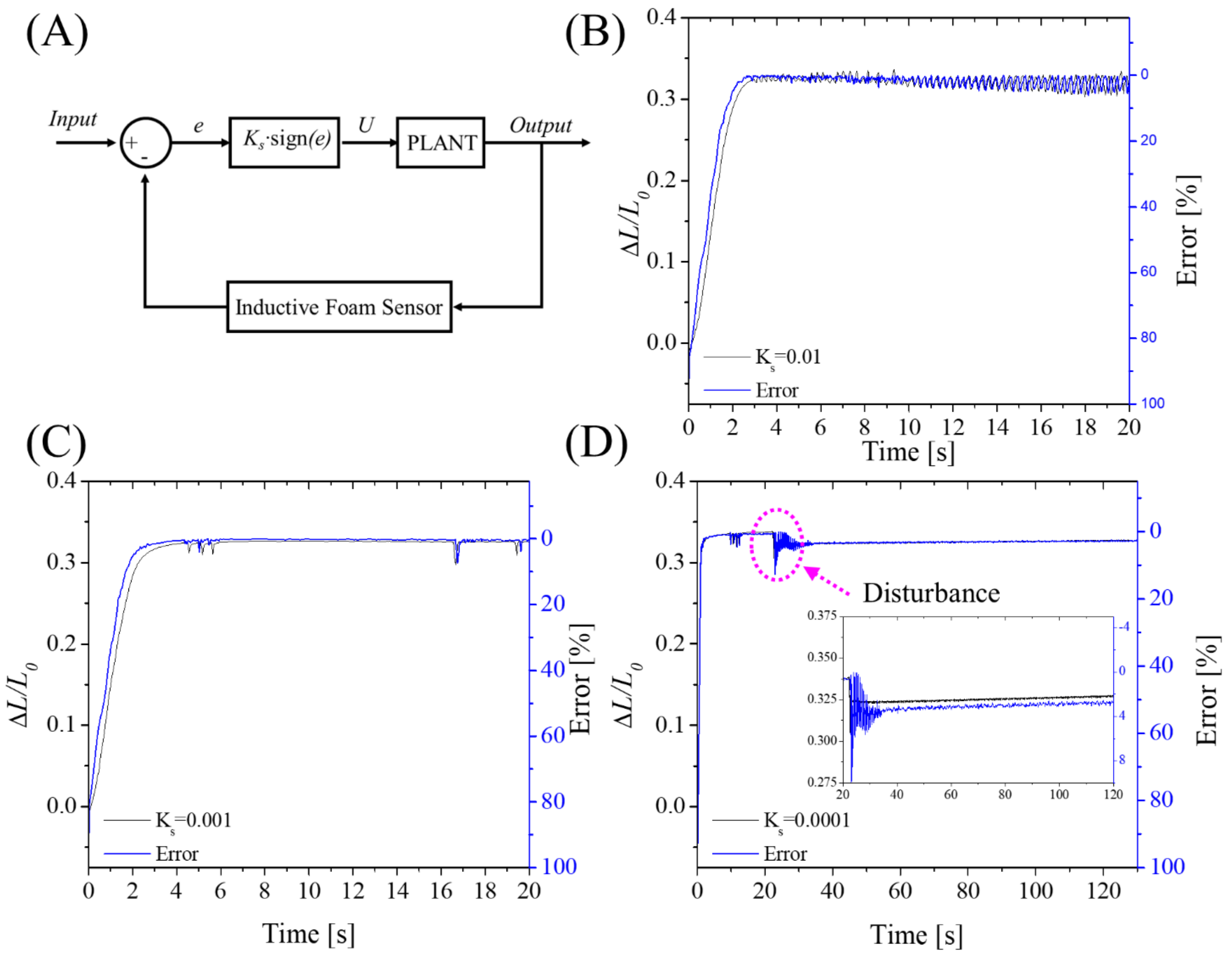

Figure S3. System configuration and response during position control. (A) block diagram of SMC, the relations of the normalized inductance and error rate when Ks is (B) 0.01, (C) 0.001 and (D) 0.0001.

Table S2. The characteristics of Sliding Mode Controller for a desired displacement

K_s constant	0.01	0.001	0.0001
Rise time [s]	1.773	1.812	0.866
Settling time [s]	22.081	17.26	1.448
Overshoot [%]	3.73	2.088	0.197

*Threshold of 5% for defining settling time.
